# 2263. Amoxicillin Use for Common Acute Respiratory Infections During a National Shortage: Results from the SHARPS-OP Benchmarking Collaborative

**DOI:** 10.1093/ofid/ofad500.1885

**Published:** 2023-11-27

**Authors:** Brian R Lee, Nicole M Poole, Joshua C Herigon, Matthew Kronman, Rosemary M Olivero, Sameer Patel, Michael J Smith, Bethany A Wattles, Ann Wirtz, Rana E El Feghaly

**Affiliations:** Children's Mercy Kansas City, Kansas City, Missouri; University of Colorado School of Medicine, Aurora, Colorado; Children's Mercy Kansas City, Kansas City, Missouri; Seattle Children's Hospital / University of Washington, Seattle, Washington; Helen DeVos Children's Hospital of Spectrum Health, Grand Rapids, MI; Ann and Robert H. Lurie Children's Hospital, Chicago, Illinois; Duke University, Durham, North Carolina; University of Louisville School of Medicine, Jackson, Mississippi; Children's Mercy Kansas City, Kansas City, Missouri; Children's Mercy Kansas City, Kansas City, Missouri

## Abstract

**Background:**

Amoxicillin is a first-line agent for most pediatric acute respiratory infections (ARIs). In Fall 2022, a nationwide amoxicillin shortage occurred. This study sought to provide precise estimates in outpatient amoxicillin usage during the shortage, using a nationally representative sample of institutions providing pediatric care.

**Methods:**

Twenty-two institutions from the Sharing Antimicrobial Reports for Pediatric Stewardship Outpatient (SHARPS-OP) Collaborative provided aggregate quarterly data from Jan 2019 - Dec 2022. Data included total counts of encounters with an ARI ICD-10 code and proportions of ARIs treated with *any* oral antibiotic, amoxicillin, and azithromycin. Data were stratified by location type (Emergency Department [ED], Urgent Care [UC], and primary care clinics [PCC]). We compared ARI prescribing (ARI encounters resulting in any antibiotic prescription) and the amoxicillin and azithromycin indices (ARI encounters resulting in amoxicillin or azithromycin prescription out of ARI encounters resulting in any antibiotic prescription) between pre-shortage (Jan 2019 - Sep 2022) and shortage (Oct 2022 - Dec 2022) time periods by location type.

**Results:**

A total 4.89 million ARI encounters (4.24 million during pre-shortage and 0.65 million during the shortage) were reported across 22 institutions. Overall antibiotic prescribing for ARI encounters changed slightly from pre-shortage to shortage period for ED (28.7% to 22.6%; delta: -6.1 [-6.4, -5.8]), UC (41.1% to 39.6%; delta: -1.6 [-1.8, -1.3]) and PCC (38.1% to 38.1%; delta: 0.03 [0.001, -0.003]). Substantial declines in the amoxicillin index were observed for ED (76.1% to 60.9%; delta: -15.1 [-15.8, -14.5]), UC (71.9% to 60.1%; delta: -11.7 [-12.1, -11.3]) and PCC (63.9% to 53.3%; delta: -10.6 [-14.2, -9.6] with considerable variation within and between SHARPS-OP institutions (Figure 1). Azithromycin use among ARI encounters decreased slightly during the amoxicillin shortage (-0.3% [-0.38%, -0.20%]).

**Figure 1:**
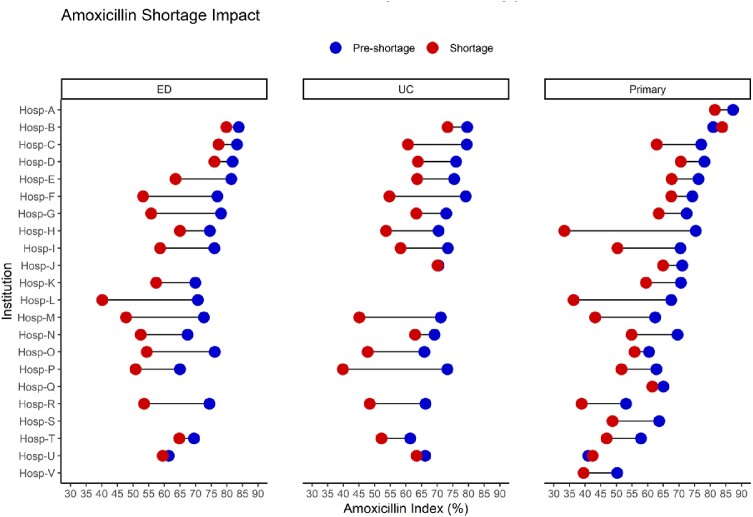
Amoxicillin indices for pre-shortage and amoxicillin shortage time periods for included SHARPS-OP institutions, stratified by location type. Location types: Emergency Department [ED]; Urgent Care [UC]; Primary Care Clinic [Primary]

**Conclusion:**

Amoxicillin utilization disproportionately decreased compared to overall antibiotic prescribing for ARIs during the shortage. Providers may have prescribed alternative antibiotics rather than no therapy for ARIs.

**Disclosures:**

**Michael J. Smith, M.D., M.S.C.E**, Merck: Grant/Research Support|Pfizer: Grant/Research Support **Bethany A. Wattles, PharmD, MHA**, Merck: Grant/Research Support

